# Supplementary material for: Ultra-low-velocity anomaly inside the Pacific Slab near the 410-km discontinuity
Source: Commun Earth Environ. 2023 May 3;4(1):149. doi: 10.1038/s43247-023-00756-y (PMC10155659; doi:10.1038/s43247-023-00756-y)
Supplement: Supplementary file 1 — Supplementary Information [file 43247_2023_756_MOESM1_ESM.pdf]

Supplementary Information

Ultra-low-velocity anomaly inside the Pacific Slab near the 410-km discontinuity

Jiaqi Li[1*,2], Thomas P. Ferrand[3], Tong Zhou[1,2,4], Jeroen Ritsema[5], Lars Stixrude[2] and Min Chen[1,6]

1: Department of Computational Mathematics, Science and Engineering, Michigan State University, East Lansing, MI 48824, USA.

2: Department of Earth, Planetary, and Space Sciences, University of California, Los Angeles, CA 90095, USA.

3: Institüt für Geologische Wissenschaften, Freie Universität Berlin, Malteserstraße 74-100, Berlin 12249, Germany.

4: Aramco Research Center, Beijing -- Aramco Asia, Beijing, 100102, China

5: Department of Earth and Environmental Sciences, University of Michigan, Ann Arbor, MI 48109, USA.

6: Department of Earth and Environmental Sciences, Michigan State University, East Lansing, MI 48824, USA.

Corresponding author email: jli@epss.ucla.edu

Supplementary Notes:

This supplementary information contains 11 figures.

Supplementary references:

1. Tao, K., Grand, S.P. & Niu, F. Seismic structure of the upper mantle beneath eastern Asia from full waveform seismic tomography. *Geochemistry, Geophysics, Geosystems* **19**(8),2732-2763 (2018).
2. White, M.C., Fang, H., Nakata, N. & Ben-Zion, Y. PyKonal: a Python package for solving the eikonal equation in spherical and Cartesian coordinates using the fast-marching method. *Seismological Research Letters* **91**(4),2378-2389 (2020).

Supplementary figures:

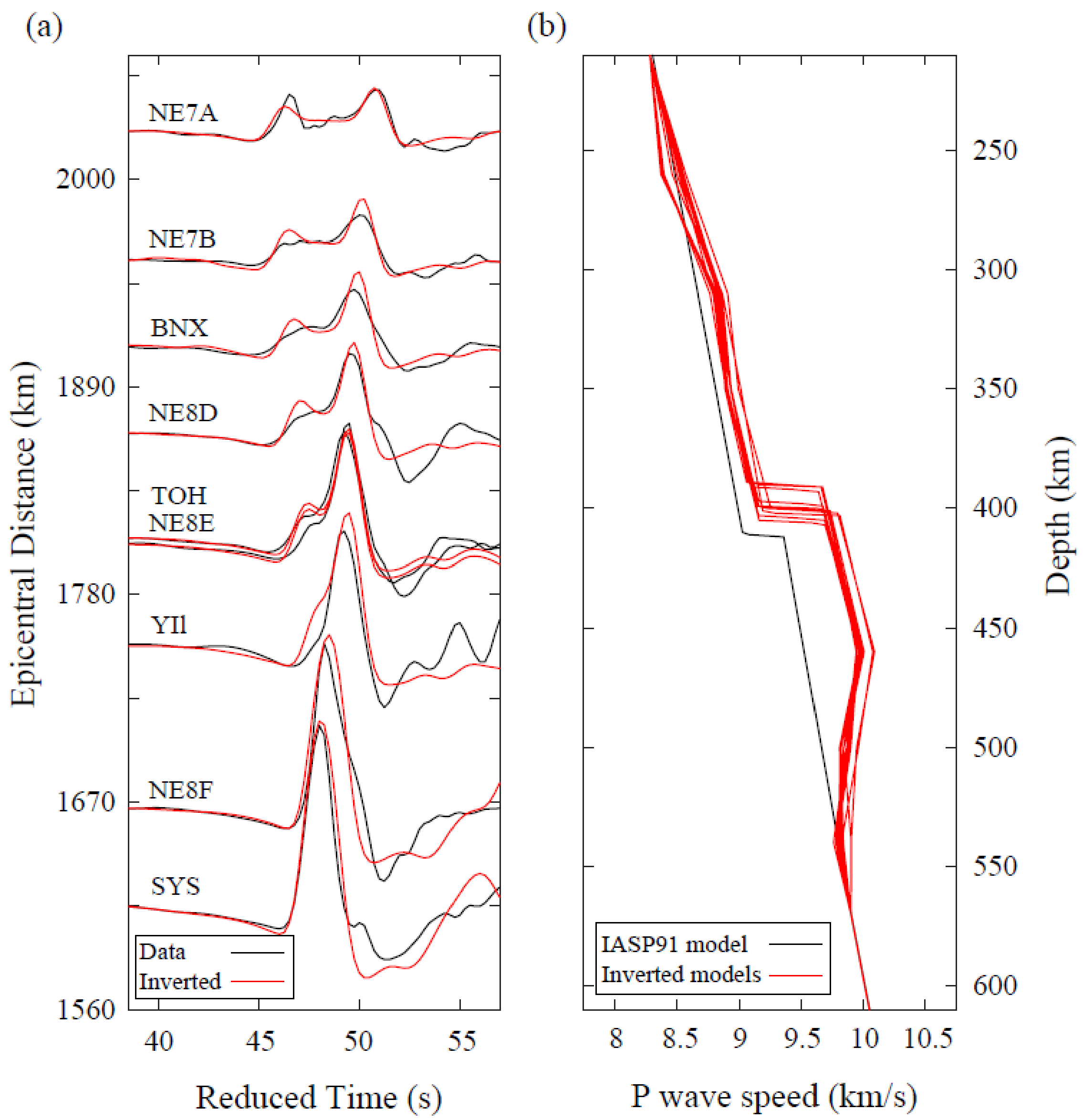

Supplementary Figure 1. 1-D Inversion results for the reference region RR'. (a) Waveform comparison between data (black) and 1-D synthetics (red). (b) Acceptable 1-D models (red).

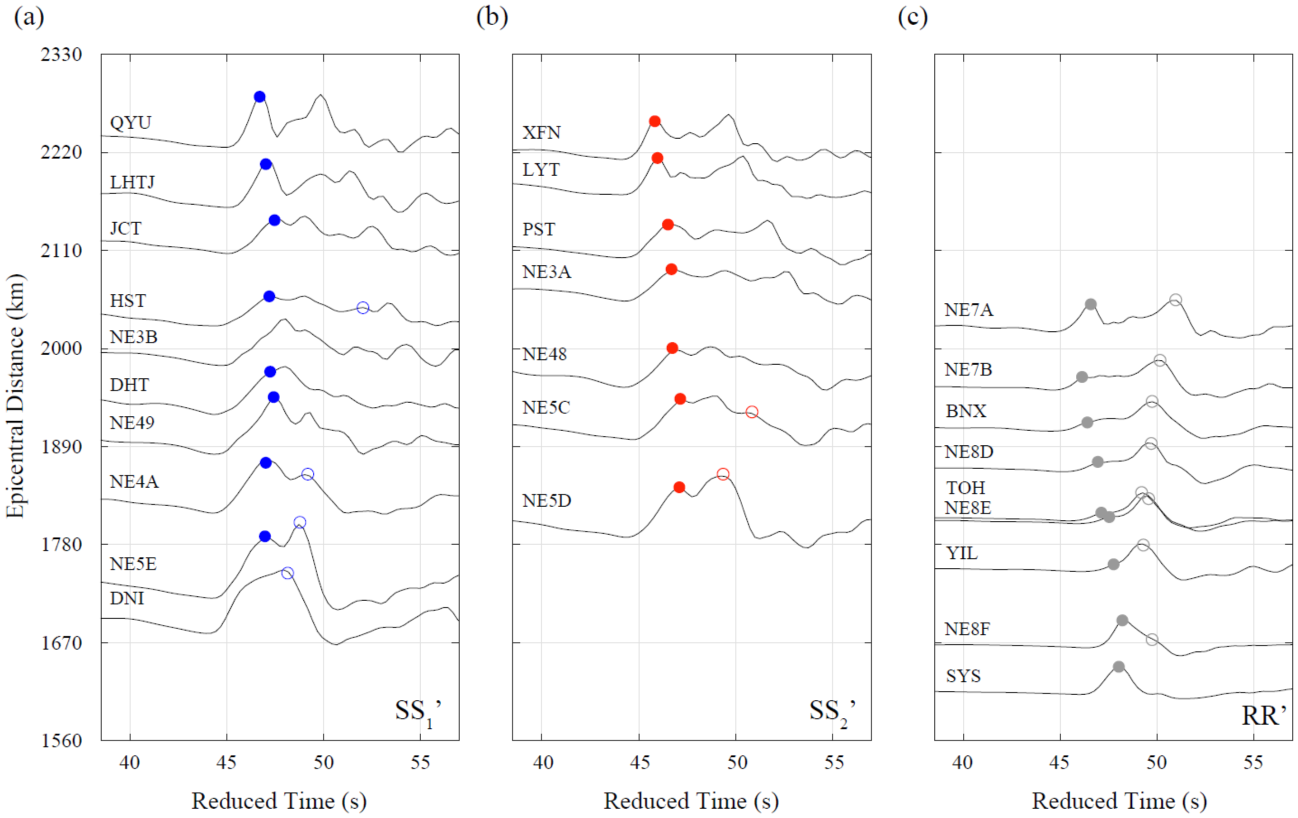

Supplementary Figure 2. Waveforms and picked arrival time. Solid and open circles mark the arrivals picked with confidence, for the refracted and direct waves, along S_1S_1' (a), S_2S_2' (b), and reference region RR' (c), respectively.

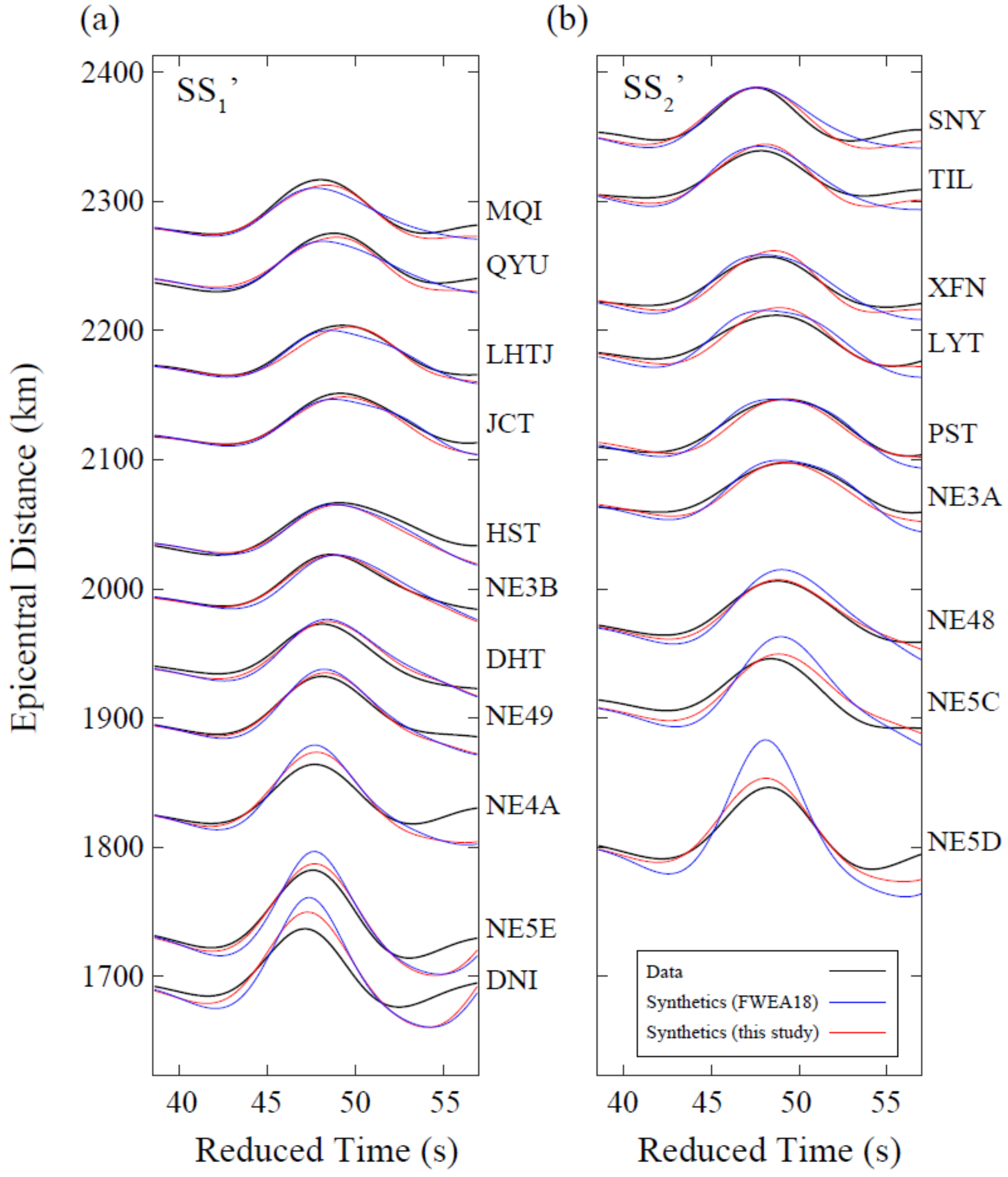

Supplementary Figure 3. Waveforms comparison at long periods. Data and synthetics are filtered from 8 to 30 seconds along SS_1' (a) and SS_2' (b). The layer out is the same as Fig. 3.

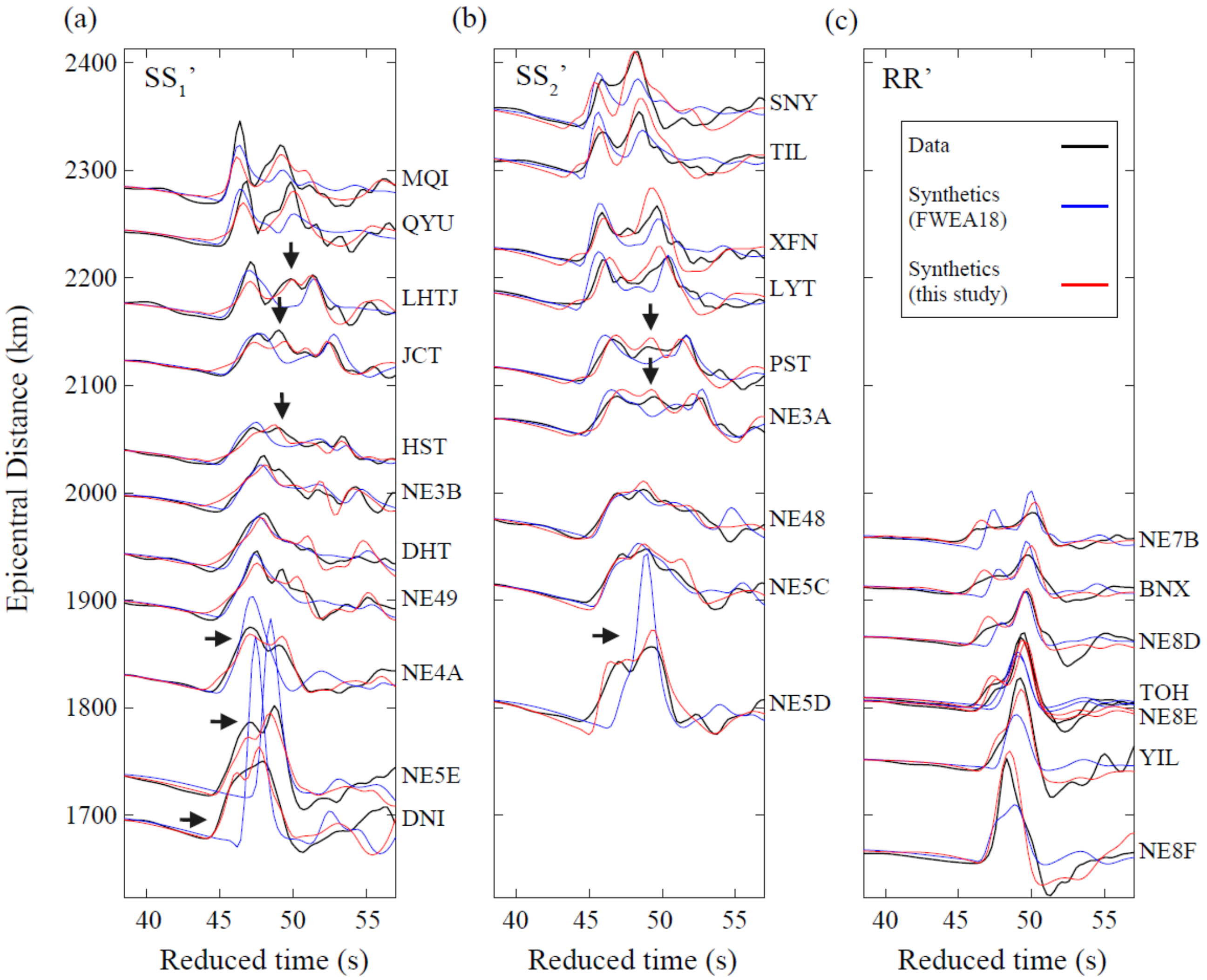

Supplementary Figure 4. (a and b) Comparison between the recorded (black) and synthetic waveforms (blue and red) for paths S1S1' (in a) and S2S2' (in b). A reduced slowness of 10.1 s/km has been applied. The blue waveforms are calculated for FWEA18[1] and the red waveforms are computed for model "FWEA18-LVZ" inverted in this study (see Fig. 4) using a finite-difference simulation code[2]. The horizontal arrows mark amplitude mismatches between the recorded and FWEA18 P waveforms. The vertical arrows mark the presence of a second P wave pulse. (c) A similar comparison for the reference path RR'.

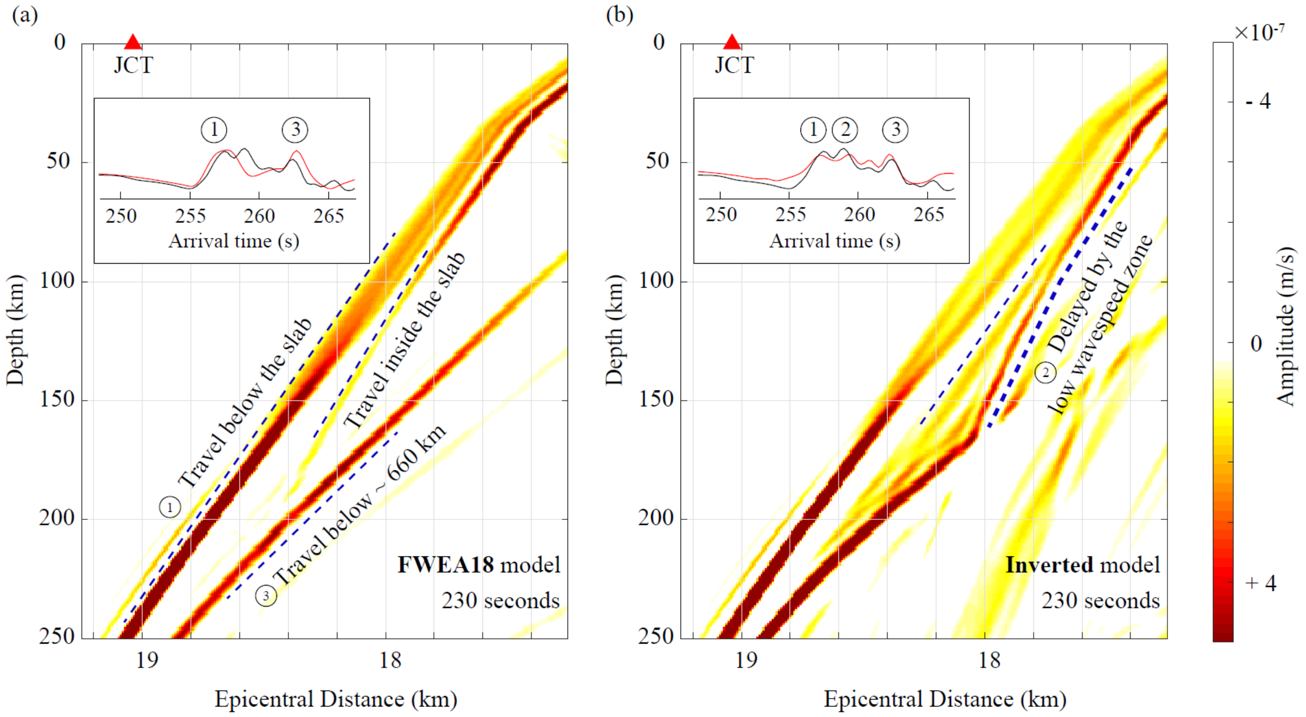

Supplementary Figure 5. Snapshot of the velocity wavefield near station JCT along SS_1'. (a) For the original model FWEA18. (b) For the inverted model "FWEA18-LVZ" in this study. Both the recorded data (black) and the synthetic waveform (red) for station JCT are shown in the upper left corner. Note the time axis here is the absolute arrival time (e.g., the second pulse arrives at ~ 258 s) rather than the reduced travel time (e.g., the second pulse arrives at ~ 49 s in Fig. 3a).

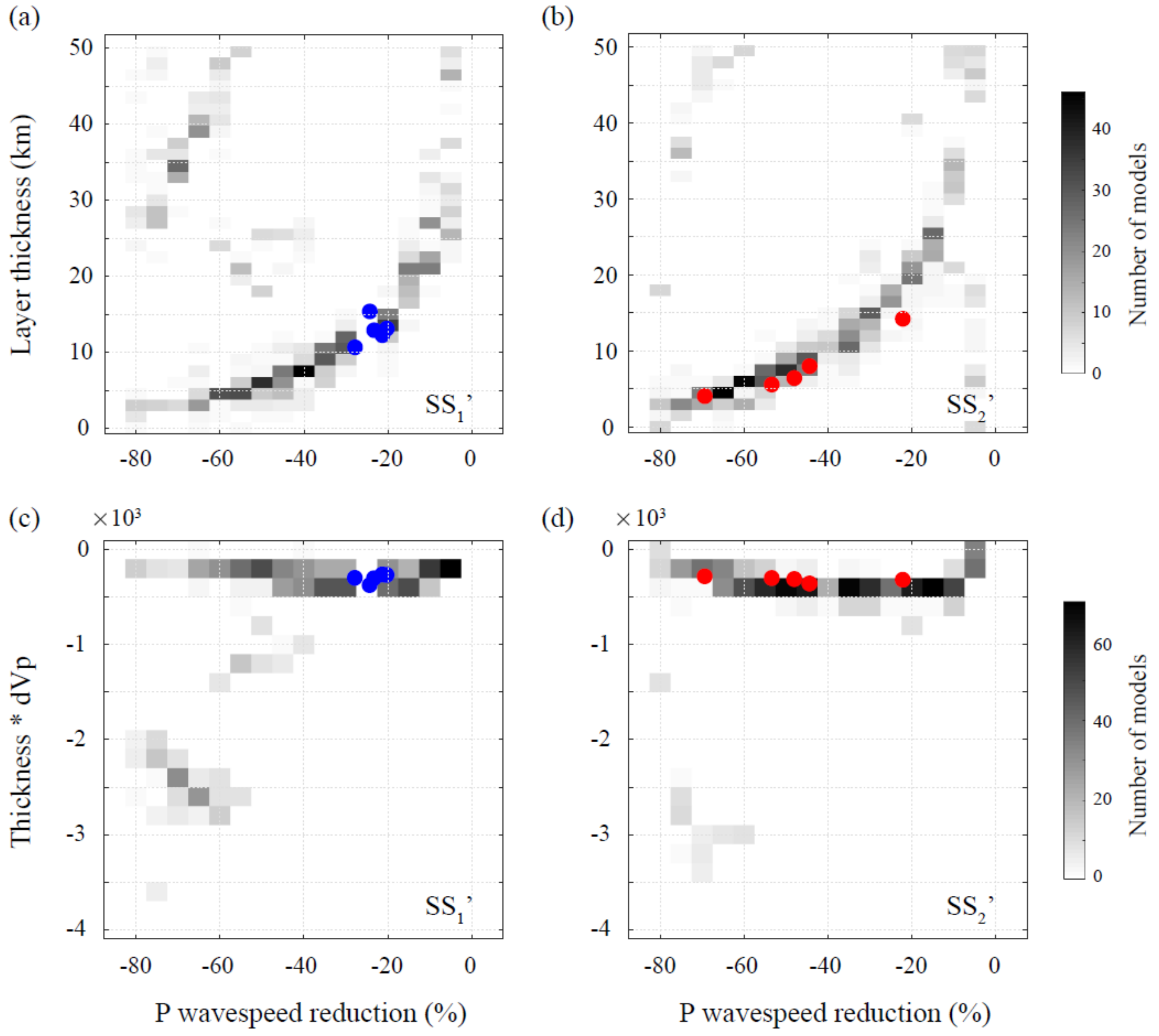

Supplementary Figure 6. Models in the last iteration. (a) P wave speed reduction and layer thickness of models in the last iteration of the inversion along SS_1'. The blue dots mark the models with the best performance (shown in Fig. 4). (c) The y-axis is the product of wave speed reduction and layer thickness along SS_1'. (b) and (d) are along SS_2', and the models with the best performance are shown as red dots.

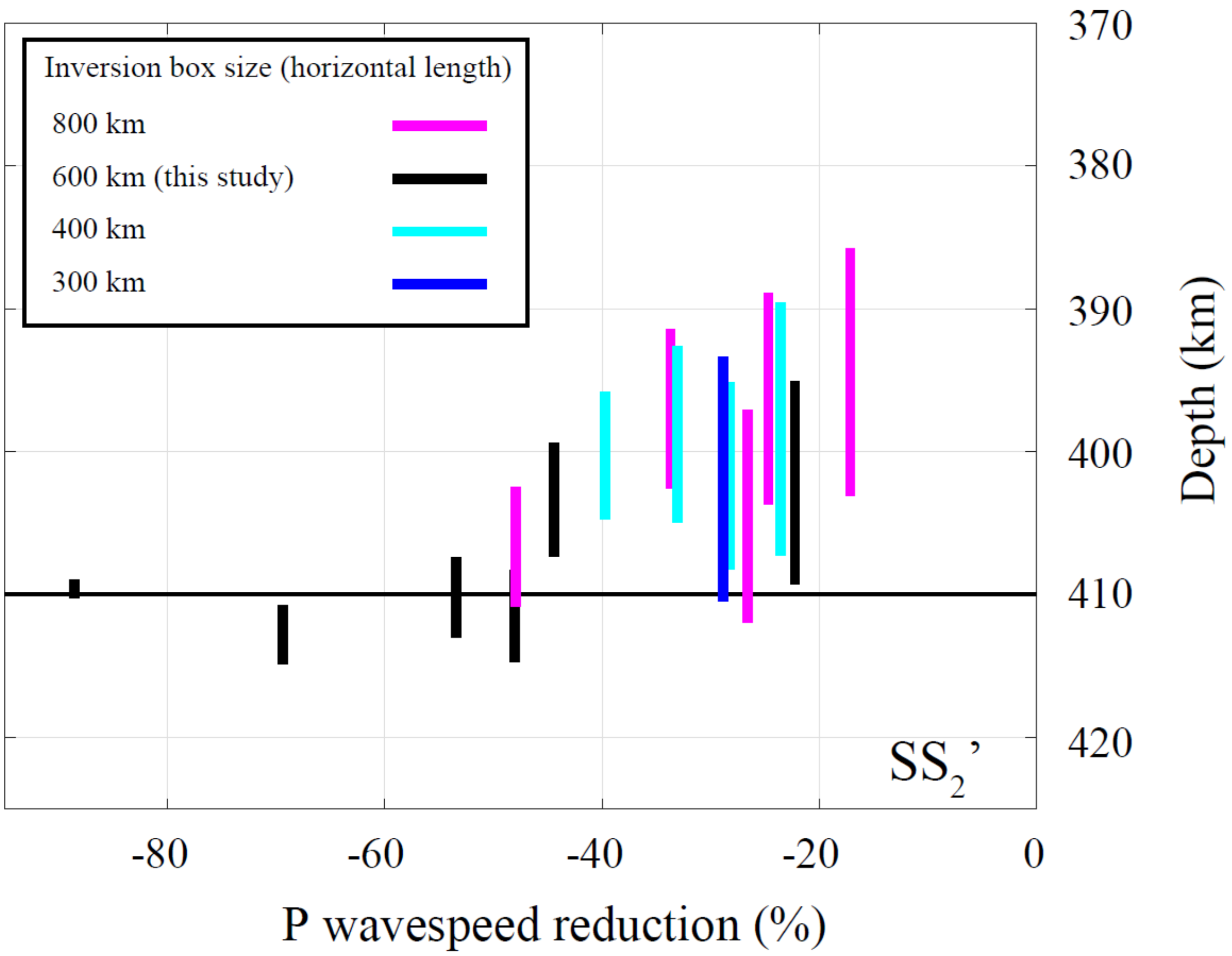

Supplementary Figure 7. Influence of the horizontal length of the inversion box. The black bars mark the acceptable models along SS_2' with a horizontal width of 600 km (used in this study). The magenta, cyan, and blue bars indicate the models for other widths of 800 km, 400 km, and 300 km, respectively. Note that when the width is smaller than 200 km, no model can fit the data.

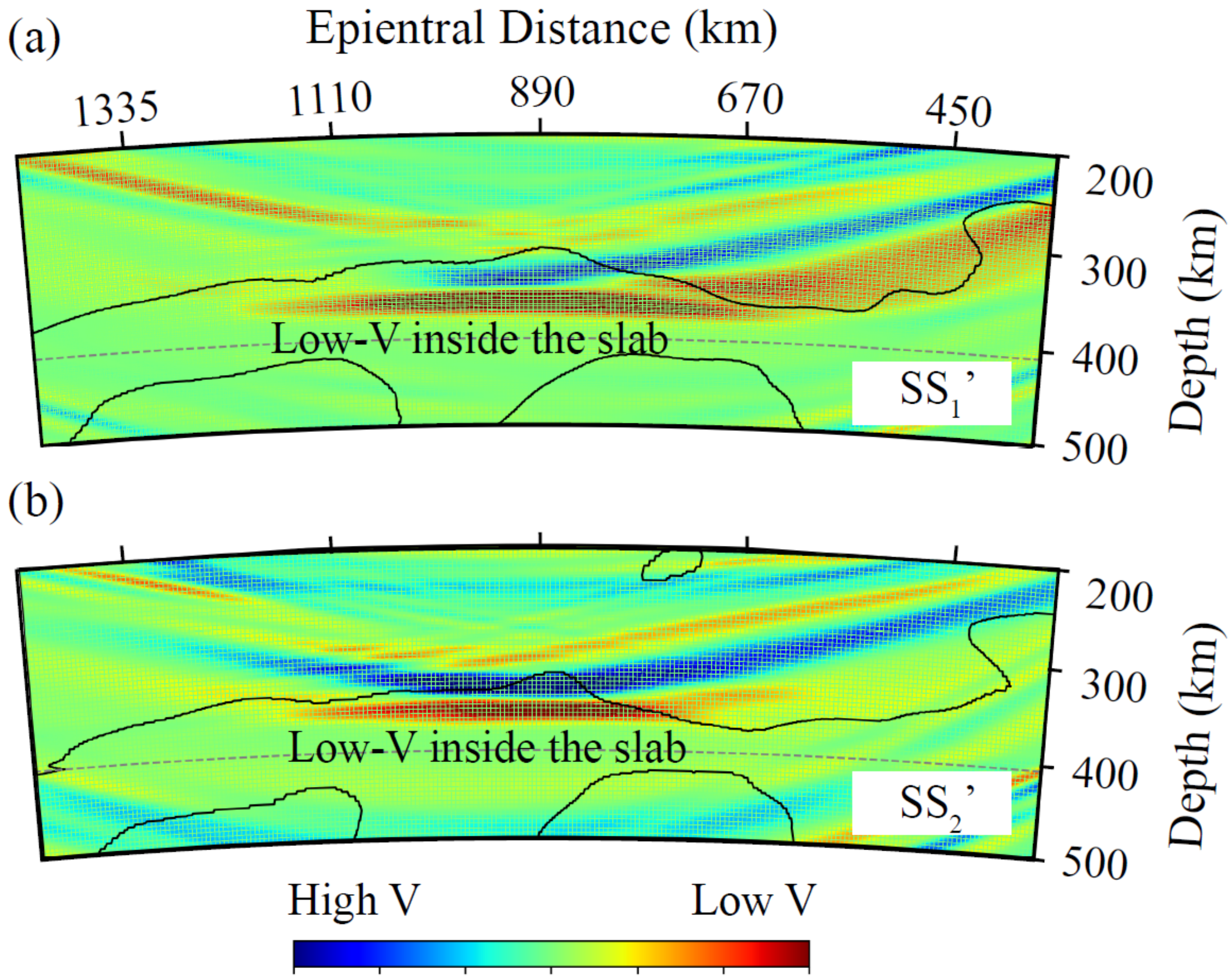

Supplementary Figure 8. Sensitivity kernels. (a) Along SS_1'. (b) Along SS_2'.

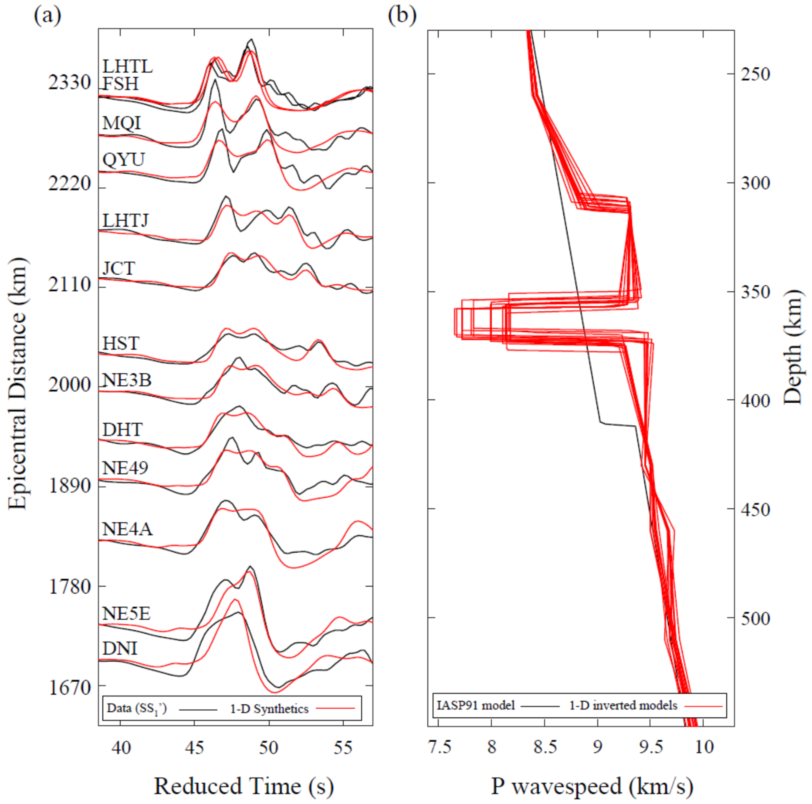

Supplementary Figure 9. Inversion results for the 1-D model along SS_1'. (a) Waveform comparison between data (black) and 1-D synthetics (red). (b) Acceptable 1-D models (red).

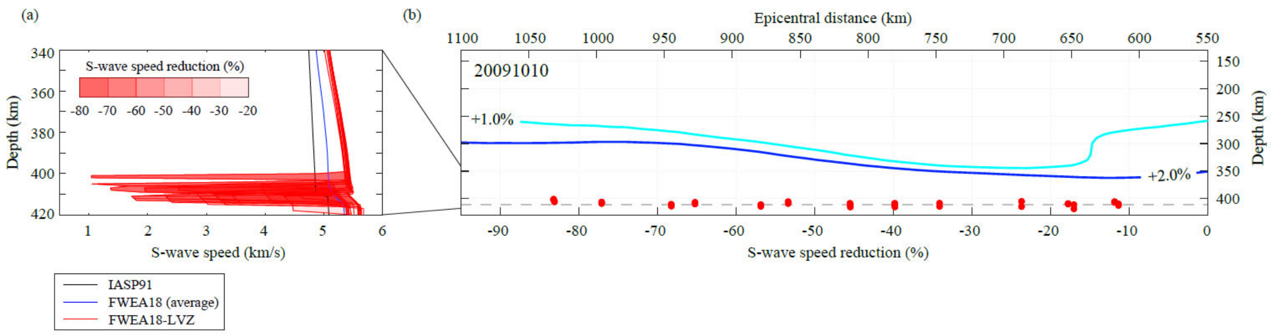

Supplementary Figure 10. Acceptable SH-wave models from waveform modeling for event 20091010 (with an azimuth range of 262°-266°). The layouts are the same as Fig. 4.

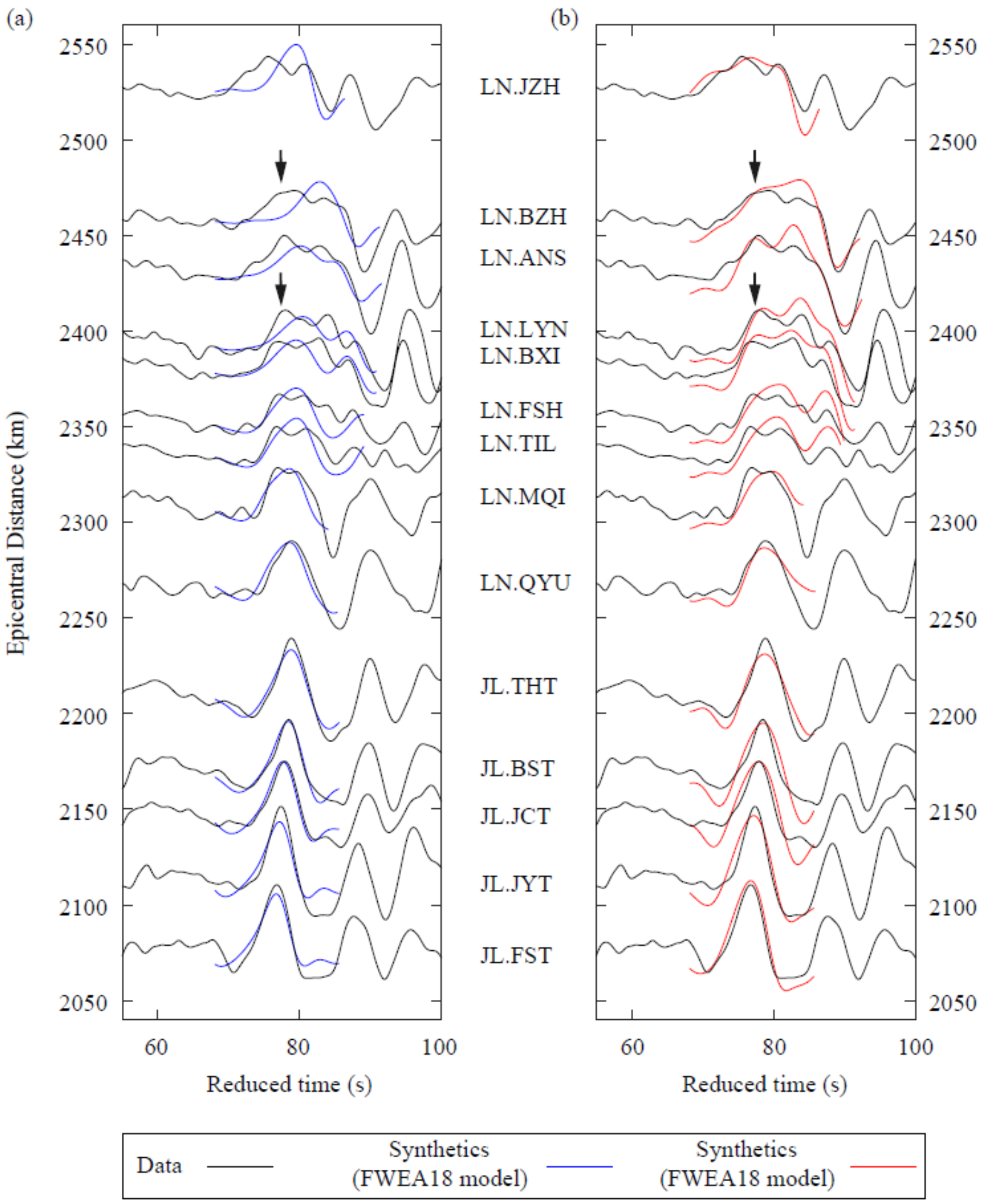

Supplementary Figure 11. Comparison between the recorded (black) and synthetic SH-waveforms (blue and red) on the tangential component for event 20091010. Waveforms are filtered between 1 s and 30 s. Note a reduced slowness of 22.0 s/km is applied to the time axis. The layouts are the same as Fig. 3.
