# Supplementary material for: Ultra-low-velocity anomaly inside the Pacific Slab near the 410-km discontinuity
Source: Commun Earth Environ. 2023 May 3;4(1):149. doi: 10.1038/s43247-023-00756-y (PMC10155659; doi:10.1038/s43247-023-00756-y)
Supplement: Supplementary file 5 — Description of Additional Supplementary Files [file 43247_2023_756_MOESM5_ESM.pdf]

Description of Additional Supplementary Files

File Name: Supplementary Data 1

Description: The seismic data generated during the analysis, i.e., both P- and S-wave displacement records after removing the instrument responses, are available as Supplementary Data 1 and also uploaded on the Zenodo repository: 10.5281/zenodo.7655059.

./20091010_RR_Pwave : P-wave data for the reference region of event 20091010.
./20091010_SS1_Pwave : P-wave data for the SS1 region of event 20091010.
./20091010_SS2_Pwave : P-wave data for the SS2 region of event 20091010.
./20090407_Pwave : P-wave data of event 20090407.
./20110804_Pwave : P-wave data of event 20110804.
./20091010_Swave : S-wave data of event 20091010.

File Name: Supplementary Software 1

Description: We put together the inversion codes used in this paper as Supplementary Software 1 and also on the Zenodo repository: 10.5281/zenodo.7655063.

This file contains the structure inversion codes.
The codes have been tested for Linux system with Tesla V100 GPU cards (NVIDIA-SMI 525.60.13 Driver Version: 525.60.13 CUDA Version: 12.0).
Those two codes could reproduce Figure 4 (the expected output)
./Example_Pwave : P-wave inversion codes, forward modeling software, data, misfit window, job submitting script.
./Example_Swave : S-wave inversion codes, forward modeling software, data, misfit window, job submitting script.
For details, please refer to: https://github.com/lijiaqi0315/FastTrip
